# Qualitative assessment of a novel results-based partnership between national wastewater surveillance centers of excellence and utility companies, Houston (Texas), Colorado, Wisconsin, and California, 2023

**DOI:** 10.1186/s12889-026-26919-y

**Published:** 2026-03-14

**Authors:** Hannah Turner, Libby Horter, Michael Welton, Cristina Martinez, Rieza Soelaeman, Kathleen Tatti, Souci Louis, Amy Kirby, Nicole Fehrenbach, John Person, Scott Santibañez, Diana Valencia

**Affiliations:** 1 U.S. Centers for Disease Control and Prevention, 1600 Clifton Rd, NE, Building 24, Atlanta, GA 30329 USA; 2 Oak Ridge Institute for Science and Education, Oak Ridge, TN USA; 3 Goldbelt Professional Services, Chesapeake, VA USA; 4 G2S Corporation, San Antonio, TX USA

**Keywords:** National Wastewater Surveillance, Utility Companies, Infectious Diseases, Surveillance, Qualitative Study, Partnerships, Centers of Excellence

## Abstract

**Background:**

The U.S. Centers for Disease Control and Prevention (CDC) initiated the National Wastewater Surveillance System (NWSS) in September 2020. Four initial Centers of Excellence (COEs) were established between 2021 and 2023 in Houston (Texas), Colorado, Wisconsin, and California to guide wastewater surveillance efforts for public health. Our objective was to increase understanding of factors that facilitated implementation of wastewater surveillance from the perspectives and experiences shared by health department COEs and wastewater utility partners.

**Methods:**

We used purposive sampling to select one key respondents from each of the four COEs and four respective wastewater utility partners. We conducted eight in-depth interviews related to the implementation of wastewater surveillance and identified common experiences and key points from interview transcription files. Insights on WWS implementation from COEs and wastewater utility partners were distilled from the responses into lessons learned.

**Results:**

Three primary themes emerged after we analyzed the interview responses: perceived community benefits from wastewater surveillance, collaboration and trust building among partners that helped program advancement, and sustainability strategies and considerations.

**Conclusion:**

This analysis provides insights into novel collaborations between utility companies and the public health sector. It highlights the need to have leadership support for program continuation and to help showcase the public health importance of WWS.

**Supplementary Information:**

The online version contains supplementary material available at 10.1186/s12889-026-26919-y.

## Background

Wastewater surveillance (WWS) existed as a public health tool prior to the COVID-19 pandemic [1]. The impact of the COVID-19 pandemic showcased the importance and utility of early detection of viruses to guide public health responses. During the COVID-19 pandemic, WWS was recognized as a cost-effective, timely, and efficient strategy to detect SARS-CoV-2 in communities and a tool to complement individual-level data offered by clinical testing [2–4]. Initial surveillance efforts monitored SARS-CoV-2 in congregate population settings and expanded to encompass surrounding communities [5–7].

The National Wastewater Surveillance System (NWSS) was established in September 2020 by the U.S. Centers for Disease Control and Prevention (CDC) to coordinate and fund WWS efforts with health departments, laboratories, and wastewater utility partners. NWSS complements traditional clinical surveillance with community-level data to detect disease trends, provides a source of data when there are low levels of clinical testing, and in some cases, serves as an early warning of increased presence of disease in communities [8]. The establishment of NWSS during the COVID-19 response has encouraged its use for other public health priorities, such as influenza, respiratory syncytial virus (RSV), mpox, enteric viruses, and antibiotic-resistant bacteria [9–12].

Four initial Centers of Excellence (COEs) based in Houston (Texas), Colorado, Wisconsin, and California were established between 2021 and 23 to serve as leaders in WWS. The COEs have partnered with utility companies and public health agencies, fostered relationships with communities they serve, and provided education and training to NWSS affiliate programs. Additionally, COEs collaborate with public health partners to advance the science of wastewater epidemiology, expand public health capacity within their agencies, and provide data that can translate into public health action [13, 14]. The perspectives shared in this study highlight the ability of these partnerships to enhance health emergency readiness and response. To inform the continued development and expansion of NWSS program, interviews were conducted with representatives from four COEs and four selected utility partners to explore sustainability, partnerships and collaboration, data use, representativeness, and other insights gained from implementation.

## Methods

We used purposive sampling to select one key respondents from each of the four COEs and four respective utility partners, resulting in eight total interviews. Each NWSS COE was represented by an expert in the WWS program within the health department and were recognized by the NWSS COEs as having extensive knowledge of the program and its implementation. NWSS COEs provided the best point of contact with experience supervising WWS activities at each utility company. Respondents were invited to participate in a single interview with an expected duration of 1.5 h over video conference call using Microsoft Teams. We developed and piloted a structured interview guide (Supplement) with questions organized based on the topic areas of representativeness, data quality and use, partnerships and collaborations, and lessons learned.

From November-December 2023, three notetakers and the same interviewer conducted seven interviews, and one respondent provided written responses via email. Verbal consent was obtained from participants, with assurances that their responses would not be linked to their names or locations. Interviews were video recorded and transcribed for data analysis. The content from transcripts and the supporting notes from each question and each responder were iteratively reviewed by three analysts and summarized by common themes and key points. Supporting quotes from interviews were identified from the transcription and organized by themes.^1^

## Results

**Fig. 1:**
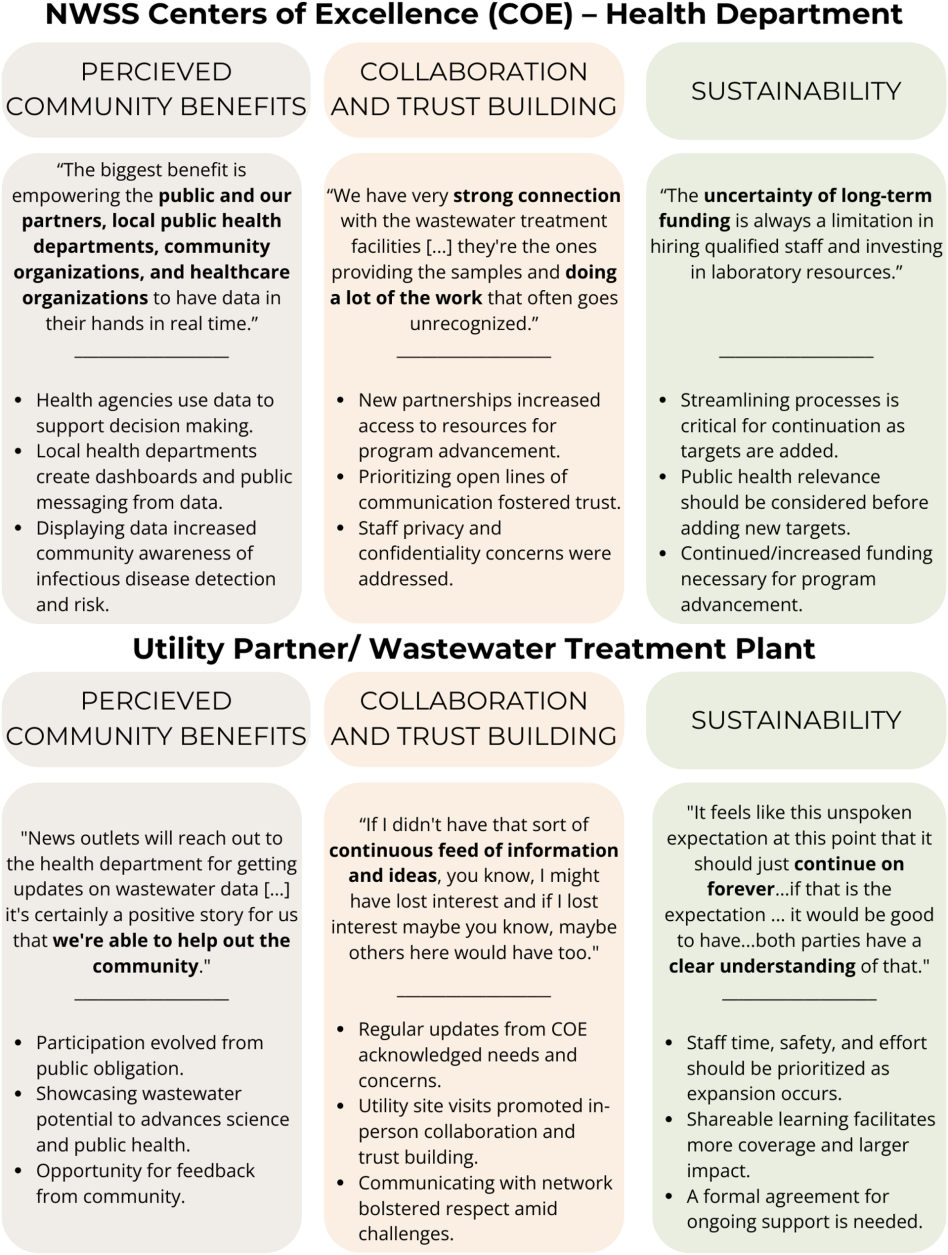
Thematic summary of respondent perspectives about the national wastewater surveillance system, California, Colorado, Houston (Texas), and Wisconsin, November – December 2023

We identified experiences related to the implementation and continuation of WWS and three common themes emerged during the analysis of the interviews 1) perceived community benefits from WWS, (2) collaboration and trust building among partners that helped program advancement, and (3) sustainability strategies and considerations (Fig. 1). Additionally, insights that COEs and utility partners shared were distilled from the responses into lessons learned described below.

### Perceived community benefits

COEs felt that WWS benefited different groups by providing reliable and timely data that empowers decision-making. A COE shared, “The biggest benefit is empowering the public and our partners, local public health departments, community organizations, and healthcare organizations to have data in their hands in real time.” COEs spoke of partnering health agencies utilizing NWSS data to make decisions related to staffing, planning, and resource allocation when infectious disease rates were high, as well as how these data supported vaccination and testing activities.

Respondents perceived that the communities served were included in NWSS processes through engagement and sharing of recommendations. COEs stated that members of their communities indicated that the public-facing dashboards have developed to become more user friendly, which has assisted with trust and reliance on the data. A COE shared:

There’s been several different counties who have adopted this really wholeheartedly and have used this as their first metric of what [disease] activity is. And so have tied their decision-making for masking and vaccines…directly to wastewater.

COEs stated that their local health departments have used WWS data to create public dashboards and messaging to share data in a timely manner. It was shared that their communities have used NWSS data posted on the public dashboards when there are increasing viral concentration trends in their area for SARS-CoV-2, influenza, RSV, and mpox. For example, one COE stated:

With mpox…more rural counties have really relied on WWS data as their only data source because testing is essentially nonexistent within their communities and so when they have detections in their areas, they can use that to really go to their health care providers and be like, hey, this isn’t just the big city problem.

The utilities stated that public health feedback and data sharing are helpful for inspiring continuation of efforts and supporting the community. One utility partner shared, “Anyone at the wastewater utility that’s involved in WWS work [is] really excited about this… they want to know what’s happening with results in their community.”

Respondents recognized that some communities are excluded from NWSS including rural and tribal communities, and other areas using septic systems and private sewages. One COE explained:

There are huge swaths of the state, both by population and landmass that are not covered, and it’s mostly these more rural communities…that are unsewered…so I’d say it’s those areas that I worry about the most because I think it’s the areas that could benefit the most from this.

COEs indicated that they are working to ensure representativeness in WWS to further program reach and promote health across communities disproportionately affected by adverse health outcomes.

Some utility partners noted that they recognized alignment of wastewater results with SARS-CoV-2, influenza, and mpox clinical case data trends shared publicly in their localities. They considered NWSS a viable tool to track pathogens. One utility partner stated, “We really get a good picture of what’s happening in that community, while not having to rely on clinical testing.”

COEs perceived that NWSS data increased community awareness of infectious disease levels and enabled assessment of risks. One COE explained, “This is another surveillance tool in our surveillance toolbox to help us inform what’s happening with disease levels and communities; it’s also anonymous.” These communities were thought to benefit from timely access to NWSS data through call centers, town hall meetings, traditional media, as well as alerts, social media, dashboards, newsletters, and email, with some materials provided in different languages. A COE stated:

News outlets will reach out to the health department for updates on wastewater data and there’s been a few stories that have happened with the press, and it’s certainly a positive story for us that we’re able to help out the community.

Some COEs noted that even though the process of information sharing has seemed to reach the intended audience, they are not always certain how the information is being used or interpreted by the public.

### Collaboration and trust building

COEs shared that having close connections was considered a factor in facilitating trust building and collaboration. One COE explained, “We have very strong partnerships with both internal and external stakeholders, and I think that’s key for a successful program.” COEs have collaborated and assisted some NWSS programs by sharing experiences and lessons learned. They have also supported other health departments with data challenges, interpretation, lab methodology questions, and ideas for public health action. A COE said, “We did train a lot of other jurisdictions on how to build their own program, they come…and see how we run our programs.” The importance of these partnerships is highlighted through advancements in modeling and data analytics, improved lab resources, and increased staffing capacity.

From the beginning, partnering utilities recognized the value of using WWS data to track infectious diseases, which facilitated the strong collaboration with the COEs. A COE stated, “Our wastewater utilities…really helped form the foundation for our whole WWS program.” The respondents from utilities indicated that their needs were considered through updates, communication, and working through problems as they arose. The connection between the utilities and the state public health department was important for developing and improving laboratory methods. Traditionally, the public health sector held a regulatory role with wastewater utilities, however this new collaborative partnership has been a hallmark of WWS. One utility partner shared:

Previously our relationship with the state was as a permittee and they were our regulator. So, it kind of took a little bit of a shift…This is the side that we’re working together, so it was a shift for sure.

Site visits to utility company facilities helped address concerns and build trust through in-person collaboration. One utility shared, “If I didn’t have that sort of continuous feed of information and ideas…I might have lost interest and if I lost interest, maybe others here would have too. And we wouldn’t be doing this.”

The ability to adapt to changing needs and consistent communication was seen to strengthen the partnerships between COEs and utilities. One utility shared:

Our communication with the [state HD] has been fantastic. They are great about sharing information with via emails. They’re great about offering different ways like they’ll offer if someone needs more clarification, they’ll offer a phone call. They’re also just really nice and pleasant to work with. So, their emails will end with like “in partnership”.

One COE has a utility relations specialist who assists utilities by providing solutions to challenges and maintaining constant communications, facilitating utility involvement. Some utility partners have benefited from small stipends to increase participation of rural or lower resourced utilities. To strengthen these connections, COEs acknowledged the value of the voluntary work that the utilities were doing. For example, one COE showcased their utility partners through a utility appreciation week, which included thank you notes, letters of appreciation, and acknowledgment on different media venues. One COE stated:

We have very strong connection with the wastewater treatment facilities and that’s so critical to the success of the program because they’re the ones providing the samples and doing a lot of the work that’s often goes unrecognized.

A utility respondent reported that WWS does not require a lot of extra work when sampling occurs at the treatment plant. They are already collecting daily samples, and participating in WWS only requires the preparation of aliquots. One utility representative shared, “I think as long as people are asking us for samples that are within our day-to-day activities that would not be a problem.” However, systems that require sub-sewershed sampling (smaller, localized areas) demand more time and effort from utilities. A utility shared:

If we had to do like sub-sewershed sampling or something more, that would be a little more complicated. [We’d need more staff]. But like I said, if a health department came to us and said, hey, we need this data, we would absolutely do what we had to do to make it happen.

Additionally, NWSS has been working to improve trust and relationships between the public health sector and the general public. One COE stated, “Things have been difficult in public health for the last few years and having this real time data source… has built that sort of public trust in public connections.” Specifically, COEs stated that prioritizing communication around ethics and data concerns has assisted in fostering trust, and that jurisdictions strive to ensure all staff, and the public are aware of the privacy and confidentiality of NWSS data. Including utilities and stakeholders in dissemination of information related to data and trends is a primary goal for the COEs. Through utilization of summary reports, meetings, and office hours, utilities and stakeholders are involved in NWSS processes and communication, stating that it is positive and supportive.

### Sustainability

Four main areas were identified by the COEs and utility partners as necessary for NWSS program to be sustainable: ethics and privacy, local authority and leadership support, continuation of funding, and collaboration and sharing experiences. First, continued efforts by NWSS to work ethically and follow privacy and confidentiality guidelines for data use and sharing were considered important for trust and sustainability. Second, having local authority and leadership support was viewed as necessary for program continuation and to help showcase the public health importance of WWS. There are no legal policies in place to support the continuation of NWSS. Health departments and the utility partners that provide the samples do not often have formal agreements pertaining to the work done. Utility partners conduct this work voluntarily and often have no company policies related to participating in NWSS. Third, the continuation of NWSS funding for program growth was noted as necessary for sustainability and considered a good return on investment. One COE stated:

The uncertainty of long-term funding is always a limitation in hiring qualified staff and investing in laboratory resources…we are working year to year on these…funding streams and makes it challenging to kind of plan out funding over the long term.

Wastewater sampling was described as less expensive than clinical testing and other surveillance systems, and one sample could be used to test for different pathogens. Fourth, COEs mentioned the importance of collaboration and sharing experiences. COEs and utilities have invested in training a workforce for NWSS and provide ongoing opportunities for continued advancement. Trainings are oriented for the different specialties that are part of WWS, such as data analytics, laboratory testing, and communications.

One COE considered that having adaptable plans of action based on subject matter expert input for new infectious disease targets contributes to sustainability. These action plans were noted to facilitate preparedness by improving communication with stakeholders and expediting program activities. COEs stated that they need to understand how the data relates to the overall population, especially when adding new targets. As the program expands to include more targets, there is a risk for surveillance not to be sustainable, due to an increase burden on laboratory staff, use of laboratory materials, and other requirements. All COEs have stated that streamlining processes is important and that they are working towards that common goal. Available protocols have allowed COEs and utilities to increase testing when needed, which has been made possible by providing testing and shipping materials to utility partners and having a dedicated courier. One utility explained:

“[state lab] really willing to do anything to make this easy for us. They send us sampling kits with everything ready to go…. They’ve sent a Courier, so we don’t even have to worry about it. A Courier comes and picks up our samples.”

### Lessons learned

While advancing WWS programs, COEs and utility partners encounter challenges. For COEs, these issues relate to wastewater data interpretation, defining indicators for public health action, improving communication among partners, and sustaining workforce capacity. Some COEs have encountered challenges when a change in the level of infectious agent or other target of interest in wastewater is above an expected threshold value, which requires analysis of the laboratory results, environmental variables, and other factors. One COE explained:

We have sewershed areas with higher tourist populations that can fluctuate from the weekday to a weekend to, you know, seasonally. So, we’re looking into alternate sources of population data to help inform our data analysis and the subsequent public health actions based on the data and we know that representativeness could be improved through alternate sources of data to inform population dynamics and through the additional analysis of sociodemographic [data].

A communication challenge is to maintain a personalized level of engagement with partners. It was noted that communication was challenging at the start of the program when attempting to identify subject matter experts and points of contact for utilities. Also, COEs stated that they need clearer communication and more collaboration among all partners and CDC. COEs and utilities agreed that despite all the communication efforts and WWS program growth, there is still space for improvement. Utilities have stated that clear expectations of their roles and a request from the health department to demonstrate why this work should continue will be important. One utility shared:

Just having clear expectations as to what our role would be, I think you know sometimes there might have been staff here that didn’t understand why it was our responsibility to do this and so having a very clear ask from public health agencies is important.

For utilities’ increased participation, they must be interested and have adequate staffing. Numerous staff members have been trained to work with NWSS, COEs noted that some staff have moved on, making continuation of activities challenging. COEs and utilities explained that due to staff turnover, there is a need for ongoing training for new hires.

NWSS program has benefited from the collaboration and commitment of the participating partners. One COE stated “We’ve been able to hire a team specific for WWS through NWSS funding. So, it has improved certainly on the lab side and on the [epidemiology] side.” COEs explained that feedback from partners and stakeholders has been crucial for improving the quality of the surveillance program. COEs have been using this feedback to improve processes related to data visualizations, trends, comparisons, and dashboards.

As the program progresses, its needs change, so continuous efforts have been made to address gaps. COEs and utilities expressed that they have adjusted to changes, while maintaining flexibility and open-mindedness. One COE stated that “The ability to pivot wastewater for new needs quickly has been really useful.” All utilities participating in WWS felt that it has been the right thing to do. A utility shared, “We felt like as a utility and having all this wonderful [influent] data available, we felt like there was something we should do. And so, as a utility, we felt obligated to do something.”

## Discussion

Cross-sector and interdisciplinary collaboration, an emphasis on building trust, and identifying and communicating community benefits were reported as driving factors for WWS success. Respondents reported factors that may support sustainability, including, establishing program-wide ethics and privacy guidelines, securing local authority and leadership support, predictable funding cycles, and continuing to expand collaboration and communication among NWSS programs. Several of these areas have been addressed in months subsequent to when these interviews were conducted, for example, the Association of State and Territorial Health Officials (ASTHO) produced the Ethics Framework for Addressing Ethical Considerations in Infectious Diseases Public Health Wastewater Surveillance with support from CDC [15].

The novel collaboration between utility partners and public health has been an essential component of NWSS. Utilities are on the front line of the surveillance system, collecting and processing the samples. Collaboration among partners was essential to the implementation of WWS during the COVID-19 pandemic and mpox response, continues to be a pillar of NWSS [13, 14, 16]. In addition to being essential for program execution, strong collaboration aids in the refinement and advancement of methodologies and analysis [17].

Several studies published since the interviews were conducted highlight the public health benefits of WWS, such as early detection of circulating diseases and improved resource allocation to assist with stopping transmission [18, 19]. These efforts have supported public health action not only in healthcare settings, but also in schools and other congregate environments [6, 20]. Respondents similarly emphasized the importance of recognizing and clearly communicating the impact of WWS. This perspective aligns with CDC’s broader efforts to foster collaboration on major health challenges, promote results-based partnerships through greater partner engagement, and create mechanisms for ongoing feedback [21]. Additionally, Centers of Excellence (COEs) emphasized the value of educating program partners—particularly those not traditionally involved in public health surveillance—which may help shift attitudes and increase program support [21].

Support from decision-makers is vital for procuring resources needed to implement and sustain public health programs [22]. Dedication from NWSS to educate and involve individuals in leadership and decision-making will help to facilitate political support for the program. A concern for WWS programs expressed in the interviews has been unpredictable funding, which has prevented long-term planning and challenges their ability to offer competitive and secure employment to qualified individuals.

The methodology used in this report allowed for rich detailed responses and insights throughout the interviews. Limitations of this methodology include the limited number of participants which could result in participant selection bias. The COE and utility partner interviewees were enthusiastic and supportive of the program, which could lead to difficulties in drawing inferences. Secondly, NWSS is a CDC funded program. The participants interviewed are those who receive funding and assistance from the CDC, which could lead to social desirability bias. However, questions were designed to be open-ended, providing each respondent with the opportunity to express both optimism about the program as well as candid critique Inclusion of such comments suggests that provided candid responses were given with regards to the best interest of the NWSS program. Lastly, the findings of this evaluation were not supported by quantitative analysis, but these findings will complement future quantitative studies which focus on the impact of WWS on public health outcomes, resource allocation, or cost-effectiveness. The results of this work, drawn from in depth conversations with individuals who possess unique and comprehensive perspectives of NWSS programs, are intended to increase understanding of NWSS collaborations and provide examples of factors that facilitated implementation.

## Conclusion

This analysis provides insights into novel collaborations between utility companies and the public health sector. It highlights the need to have leadership support for program continuation and to help showcase the public health importance of WWS.

## Supplementary Information

Supplementary Material 1.

## Data Availability

The datasets used and/or analysed during the current study are available from the corresponding author on reasonable request.

## Footnotes

1 This activity was reviewed by CDC and was conducted consistent with applicable federal law and CDC policy.§ (§See 45 C.F.R. part 46, 21 C.F.R. part 56; 42 U.S.C. Section  241(d); 5 U.S.C. Sect. 552a; 44 U.S.C. Section  3501 et seq).

## Abbreviations

CDC: Centers for Disease Control and Prevention
COE: Centers of Excellence
HD: Health department
NWSS: National Wastewater Surveillance System
RSV: Respiratory syncytial virus
WWS: Wastewater Surveillance
