# Supplementary material for: Qualitative assessment of a novel results-based partnership between national wastewater surveillance centers of excellence and utility companies, Houston (Texas), Colorado, Wisconsin, and California, 2023
Source: BMC Public Health. 2026 Mar 14;26:1314. doi: 10.1186/s12889-026-26919-y (PMC13101207; doi:10.1186/s12889-026-26919-y)
Supplement: Supplementary file 1 — Supplementary Material 1. [file 12889_2026_26919_MOESM1_ESM.docx]

| ***Interview Script for COEs*** | |
| --- | --- |
| Representativeness | |
| Did you have wastewater surveillance in your jurisdiction before this funding opportunity? | |
| As a NWSS Centers of Excellence what can you contribute to other jurisdictions for them to become CoEs? | |
| What is the immediate benefit of WWS for your jurisdiction? | |
| Have communities in your jurisdiction benefited from wastewater surveillance efforts? Please explain. | |
| Are there any parts of the community that are not represented by the NWSS in your jurisdiction that you are aware of? Have efforts been made to address gaps in data among unsewered areas? | |
| Has the public health capacity been improved in your jurisdiction by wastewater surveillance efforts? Please explain. How do you think it can be further improved? **(public health capacity - (new staff, trained staff, equipment increase)** | |
| [Post pandemic?] Have public health actions been taken based on findings from wastewater surveillance? Or will be taken in the future? Please explain. | |
| Data Quality and Use | |
| How well does the data collected represent your jurisdiction? Have you made any innovations to be more representative? How can representativeness of data be improved? | |
| Are the NWSS data communicated back to the community? Please explain. What method has worked best? What could be improved/challenges? | |
| Have you taken any steps to make data sharing and reporting more accessible for your community? **(people with no computer access, or no social media)** | |
| Has your data management **(collection, cleaning, sharing)** for wastewater surveillance changed **(and in what way?)** in your jurisdiction? | |
| Has a NWSS point person been identified for data-related questions? How has this worked or not worked? | |
| Are hotspots identified and monitored? What steps for automation of hotspot identification/monitoring have been made? | |
| Have steps been taken to standardize wastewater testing in your jurisdiction? How has this effort been led? What is the progress so far? What else can be done to improve? | |
| Have you encountered in ethical concerns or questions regarding use of wastewater data? ...from the public? From partners? | |
| Partnerships and Collaboration | |
| What partners are included in your jurisdictions to ensure that community members are represented in and informed on the NWSS? | |
| What has been your local experience communicating with partners (utility or HD or CDC)? What improvements have been made? What more can be done to improve communication? | |
| Can you tell us about the communication strategies for people that can’t use a dashboard or are not using social media? | |
| Are there any local policies in place to support the NWSS? **(regulation/guidelines in your jurisdiction)** | |
| Can you tell us about your experience with the contracting company that collects the samples? (any challenges) | |
| How are utility partners retained and engaged? | |
| Has your CoE prepared any products for the utility company that showcase results based on their wastewater sampling? | |
| Can you tell us about your experience with the lab contracting company that processes the samples? **(any challenges)** | |
| Do you know if there have been any secondary impacts of NWSS funding beyond wastewater surveillance? (For example, has lab equipment funded by NWSS also been useful for other testing?) | |
| Anything else you want to share about your partners? **(Concerns or challenges?)** | |
| Lessons Learned and Expectations | |
| What are the strengths of your jurisdiction in terms of wastewater surveillance? | |
| What are the challenges of your jurisdiction in terms of wastewater surveillance? | |
| How sustainable is wastewater surveillance in your jurisdiction? **(without NWSS funding, will your jurisdiction be able to continue doing WWS?)** What can be done to make it more sustainable? (policies/legislation in place) | |
| What future expectations do you have for continuation and expansion of the National Wastewater Surveillance System? | |
| What would continuation of funding mean for the NWSS? | |
| What could be done differently to improve the system or that could increase the chances that this system will continue to be useful? | |
| Have there been any public concerns regarding NWSS in your jurisdiction? | |
| Is there anything else that we should know about your surveillance system that you would like to share? | |
| **Interview Script for CoE Utility Partners** |  |
| Partnerships |  |
| Do you know the reasons why your company decided to participate in this project? If yes, please tell us about that. |  |
| Is your participation with this project voluntary? |  |
| Have you worked with the health department previously or is this a new partnership? |  |
| When was your partnership created with "xx" health department in regard to this project? |  |
| What is your communication and relationship with the health department about wastewater sampling? |  |
| What is your communication and relationship with the lab that processes the samples? |  |
| What type of information is shared with the Health Department or the labs? (number of samples? How often and how are they shared? Etc…) |  |
| Does the utility company provide any public health messaging to the communities regarding this project? For example in newsletters or on the bill statement? |  |
| Are there other organizations or outside partners other than the health department that you work with on this project? |  |
| Logistics |  |
| Do you feel your program is properly staffed to support wastewater sampling? |  |
| How many staff members are working on this project? |  |
| What type of training did your staff receive before starting this project? Can you describe the topics of the training? How often do the staff get trained? |  |
| What safety measures are in place to ensure staff are protected while collecting samples? |  |
| Have staff expressed any concerns with conducting surveillance on pathogens? |  |
| Please describe turn around in the staff. If staff turnover is a challenge, what do you think could be done to maintain staff? |  |
| Who provides the supplies for sampling? Do you feel your program is adequately supplied? |  |
| Sampling |  |
| Do you know about how much of the population **your** company covers for this program? Please explain. |  |
| How many sites are sampled for this project? |  |
| Are there plans to increase/improve your coverage of sampling? |  |
| How are the samples collected? |  |
| How often do you sample the wastewater for this project? |  |
| How often are the samples shipped to the lab? |  |
| Does your utility company hold the samples before sending to the lab? If yes, for how long? If yes, what are the reasons for holding the samples? |  |
| What steps have been taken to help improve timeliness between sampling and sending to the lab? |  |
| Does your utility company do any onsite testing? Or are all samples sent to the lab? |  |
| Sustainability and Future Expectations |  |
| Does your utility company plan to continue supporting these efforts? If yes, how; if no, why |  |
| Are there any policies in your company that are in place that could support continuation of this project? |  |
| If wastewater sampling expanded, what would be your company's perspective, in other words, would the company be able to support it, would it be feasible, challenging, etc? |  |
| Lessons Learned |  |
| What do you think is the main benefit of your company’s participation in this project? |  |
| What have been successes in your program since implementing wastewater sampling? |  |
| What has been challenging in your program since implementing wastewater sampling? |  |
| Have you received any positive feedback from the community? Please explain. |  |
| Have you received any negative feedback or concerns from the community? If yes, what, and how did your company respond |  |
| Are there any standardized processes that your company utilizes that you think could be useful for other sites? |  |
| For utility companies that may be hesitant to participate in WWS, is there anything you would say to encourage them? |  |
| Is there anything that could be done to improve your experience with the work you do? |  |
| Is there anything else you would like to share with us? |  |
